# Histidine Enhances the Anticancer Effect of Gemcitabine against Pancreatic Cancer via Disruption of Amino Acid Homeostasis and Oxidant—Antioxidant Balance

**DOI:** 10.3390/cancers15092593

**Published:** 2023-05-03

**Authors:** Narendra Kumar, Satyanarayana Rachagani, Gopalakrishnan Natarajan, Alexandra Crook, Thiyagarajan Gopal, Vinothkumar Rajamanickam, Jyoti B. Kaushal, Sirpu N. Nagabhishek, Robert Powers, Surinder K. Batra, Viswanathan Saraswathi

**Affiliations:** 1 The Department of Internal Medicine, Division of Diabetes, Endocrinology and Metabolism, University of Nebraska Medical Center, Omaha, NE 68198, USA; 2 The VA Nebraska Western Iowa Health Care System, Omaha, NE 68105, USA; 3 Department of Biochemistry and Molecular Biology, University of Nebraska Medical Center, Omaha, NE 68198, USA; 4 The Department of Chemistry, University of Nebraska-Lincoln, Lincoln, NE 68588, USA; 5 Nebraska Center for Integrated Biomolecular Communication, University of Nebraska-Lincoln, Lincoln, NE 68588, USA

**Keywords:** pancreatic cancer, histidine, gemcitabine, glutathione, hydrogen peroxide, metabolomics

## Abstract

**Simple Summary:**

Amino acid metabolism is aberrantly altered in pancreatic cancer (PC). Evidence suggests that circulating histidine (His) levels are low in PC. However, the role of His in modulating the progression of PC remains unknown. We determined the role of His in altering the therapeutic effectiveness of gemcitabine (Gem) against PC. We provide evidence that the expression of histidine ammonia lyase (HAL), an enzyme involved in His catabolism, is greatly increased in mouse and human pancreatic tumors. We show that combining His with Gem led to enhanced cytotoxic effects against aggressive PC cell lines. Our metabolomics analysis revealed that His treatment results in the depletion of amino acids supporting glutathione production. His depletes cellular glutathione and increased hydrogen peroxide production, indicating that His potentiates the cytotoxic effects of Gem by promoting oxidative stress. A combination of His and Gem was effective in attenuating pancreatic tumor growth and improving the survival of mice exhibiting xenograft tumors. Our findings suggest that His supplementation is an effective nutritional approach to enhance the efficacy of chemotherapeutic drugs.

**Abstract:**

Due to the severe toxicity posed by chemotherapeutic drugs, adjuvant nutritional intervention has gained increased attention in the treatment of pancreatic cancer (PC). Amino acid (AA) metabolism is aberrantly regulated in PC and circulating histidine (His) levels are low in PC patients. We hypothesized that His uptake and/or metabolism is dysregulated in PC and that combining His with gemcitabine (Gem), a drug used in the treatment of PC, will enhance the anti-cancer effects of Gem. We performed in vitro and in vivo studies to determine the anticancer effect of the combination of His and Gem against lethal PC. We demonstrate that circulating His levels are low in both human subjects and genetically engineered mice exhibiting pancreatic tumors. Interestingly, the expression of histidine ammonia lyase, an enzyme involved in His catabolism, is higher in PC compared to normal subjects. His + Gem exerts a more potent cytotoxic effect in PC cells compared to individual treatments. His treatment results in a profound increase in His accumulation, accompanied by a depletion of a number of AAs, promoting cancer cell survival and/or glutathione (GSH) synthesis. His but not Gem increases hydrogen peroxide and depletes cellular GSH. Supplementation with GSH protects cells against His + Gem-induced cytotoxicity. Further, our in vivo studies demonstrate that His + Gem potently reduced tumor mass and improved mouse survival. Taken together, our data suggest that PC cells exhibit an aberrant His uptake/accumulation which, in turn, leads to oxidative stress and depletion of AA pool, thereby enhancing the anticancer effect of Gem.

## 1. Introduction

Pancreatic cancer (PC) is projected to be the second leading cause of cancer-related death by the 2030s [1]. Current therapies have only a modest impact on survival. Moreover, these drugs are toxic to normal cells and often become ineffective due to chemoresistance resulting in treatment failure. It is becoming clear that metabolic disorders such as obesity and type 2 diabetes greatly increase the risk of PC [2,3,4], highlighting the role of altered metabolic pathways in the pathogenesis of this devastating disease.

Gemcitabine (Gem), the first-line chemotherapeutic agent for PC, provides symptomatic improvement only in a small proportion of patients [5]. Although the new combination therapy using oxaliplatin, irinotecan, leucovorin and 5-fluorouracil showed improved response compared to Gem alone, there was a significant toxicity in PC patients [6]. Of note, Gem is frequently used in combination therapy which has been shown to have an enhanced therapeutic benefit in several studies [7,8,9]. Although several combination approaches are employed to treat PC, it remains a highly malignant tumor with poor prognosis and low survival rate. Of several chemotherapeutic drugs used to treat PC, Gem exhibits better tolerance and safety. Because cancer cells exhibit rewired metabolic networks, the nutritional approach is gaining increased importance in the treatment of many types of cancer. Therefore, identifying ways to enhance the effectiveness of Gem with a nutritional component would be a safe approach to effectively destroy cancer cells. It is becoming increasingly evident that altered amino acid (AA) metabolism is a hallmark of PC. For example, glutamine is a preferred metabolic substrate for PC [10]; moreover, the branched-chain AAs are involved in modulating the progression of PC [11]. However, the role of other AAs in altering PC growth is unclear.

Emerging evidence suggests that histidine (His) uptake and/or metabolism is altered in PC. For example, previous metabolomics studies showed that His levels are low in PC [12,13]. Although an association exists between low circulating His and PC, potential mechanisms by which His modulates the progression of PC is unclear. Histamine, derived via histidine decarboxylase, has been shown to inhibit the growth of PC [14]. However, histamine regulation of PC is highly complicated and ambiguous due to the differential effects of different histamine receptors in modulating cell proliferation [15]. It should be pointed out that deamination of His via histidine ammonia lyase (HAL) is the first step involved in His catabolism. Importantly, it has been reported that HAL expression is associated with methotrexate sensitivity in cancer cell lines and with survival rate in patients [16]. Moreover, in vivo dietary supplementation of His increased flux through the His degradation pathway and enhanced the sensitivity of leukemia xenografts to methotrexate. However, the potential of His supplementation in altering the therapy response to PC and the potential mechanisms remain unknown. Because circulating His levels are low in PC patients and His supplementation improves the efficacy of certain anticancer drugs, we hypothesize that His uptake and/or metabolism is aberrantly regulated in PC and that targeting His catabolism will be an effective strategy to enhance the anticancer effects of Gem.

Using in vitro and in vivo models, we demonstrate that His in combination with Gem exerted an enhanced anticancer effect. Our studies in cultured PC cells provide evidence that a combination of His and Gem led to an enhanced cytotoxic effect than either treatment alone. The improved therapeutic benefit offered by the combined treatment is mediated via an aberrant uptake and accumulation of His and an imbalance between oxidants and antioxidants. Our detailed metabolomics analysis provides evidence that His supplementation results in the depletion of many AAs involved in glutathione (GSH) production. Our in vivo studies in orthotopic mouse models of PC further demonstrate that a high His (HH) diet + Gem therapy inhibited pancreatic tumor growth and led to an improvement in mouse survival. Taken together, our data suggest that His supplementation is a promising nutritional approach in enhancing the therapy response against lethal PC.

## 2. Materials and Methods

### 2.1. Cell Lines

Human PC cell lines SW1990 and Colo357 were obtained from American Type Culture Collection. Normal pancreatic cell line [human pancreatic nestin expressing (HPNE)] was a kind gift from Dr. Michel Ouellette at UNMC. Cells were cultured in Dulbecco’s Modified Eagle Medium (DMEM), 10% fetal bovine serum (FBS) and 100 units/mL penicillin and 100 μg/mL streptomycin. Cells were treated with amino acid-free DMEM-F12 media containing 5% FBS unless otherwise indicated. For orthotopic implantation of PC cells to develop xenograft and syngeneic mouse models of PC, SW1990 cells and murine PC cells (UN-KPC-960, established at UNMC) were used, respectively. A detailed list of cell lines, media, mouse models, and other materials used for this project is provided in Supplemental Table S1.

### 2.2. MTT Assay

The cytotoxicity of His and Gem against SW1990 and Colo357 cells was measured by MTT (3-(4,5-dimethylthiazol-2-yl)-2,5-diphenyltetrazolium bromide) assay. Cells were seeded in 96 well plates using DMEM containing 10% FBS. After 70–80% confluency, cells were treated with His (up to 25 mM) and/or Gem (10 µM) using AA-free DMEM F-12 with 5% FBS for up to 72 h. After treatment, MTT (5 mg/mL) was added, and cells were incubated for 4 h at 37 °C. Cell culture supernatant was discarded, and formazan crystals were dissolved in dimethyl sulfoxide (DMSO). Plates were read at 570 nm.

### 2.3. Presto Blue Cell Viability Assay

Presto blue cell viability assay was performed according to the manufacturer’s instructions (Thermo Fisher Scientific, Waltham, MA USA). Briefly, PC cells were seeded in 96 well plates at a density of 5000 cells per well and incubated at 37 °C overnight. Cells were treated with appropriate test compounds. At the end of the treatment period, 1× Presto blue was added and cells were further incubated for 10 min at 37 °C and the cell viability was determined using a fluorescent plate reader at an excitation wavelength of 560 nm and emission wavelength of 590 nm. Relative cell viability was determined as the percentage compared with control.

### 2.4. Metabolomics Analysis

Samples were prepared, and NMR and statistical analyses followed our previous publication [17]. Briefly, SW1990 and Colo357 cells were treated with 25 mM His and/or 10 μM Gem for 24 h. Isotopically labeled experiments were treated with 5 mM [^13^C^15^N]- labeled His and 10 μM Gem for 2D NMR analysis. Metabolites were extracted with methanol and water, dried by lyophilization, and reconstituted in 50 mM pH 7.2 phosphate buffer in 99.8% D_2_O prior to NMR analysis.

A 50 μM or 500 μM solution of TMSP was used as an internal standard for one-dimensional (1D) ^1^H NMR or two-dimensional (2D) NMR analysis, respectively. 1D ^1^H and 2D ^1^H-^13^C HSQC experiments were collected on a Bruker AVANCE III-HD 700 MHz spectrometer at 298 K using a 5 mm QCI-P inverse quadruple-resonance (^1^H, ^13^C, ^15^N, ^31^P) cryoprobe with a *z*-axis gradient, a SampleJet, an autotune and match system. Metabolites from 1D ^1^H NMR spectra were identified by Chenomx (Edmonton, AB, Canada). 2D ^1^H-^13^C HSQC spectra were processed with NMRpipe [18], and metabolites were identified using HMDB [19], BMRB [20], and a chemical shift uncertainty of 0.08 ppm ^1^H and 0.25 ppm ^13^C. A total of 96 1D ^1^H spectra and 64 2D ^1^H-^13^C HSQC were acquired with 6 and 4 replicates per group, respectively. Spectra were processed with one zero-fill, and as needed, aligned with Icoshift [21], binned using the intelligent adaptive binning algorithm [22], scaled with UV scaling, and normalized with standard normal variate for principle component analysis (PCA) or orthogonal projections to latent structures (OPLS). OPLS models were validated with CV-ANOVA and a permutation test (*n* = 1000).

### 2.5. Amplex Red Hydrogen Peroxide Assay

Hydrogen peroxide (H_2_O_2_), one of the highly unstable reactive oxygen species (ROS), was measured using the Amplex Red hydrogen peroxide/peroxidase assay kit (Invitrogen). Briefly, PC cells seeded in 12-well plates were treated with His and/or Gem for 72 h. At the end of the treatment period, media samples were collected and used for the assessment of H_2_O_2_. Briefly, 50 µL of experimental media and H_2_O_2_ standards (0, 1, 2, 3, 4, and 5 µM concentrations) were loaded in duplicates in the 96-well microplates. To initiate the reactions, 50 µL of Amplex Red working reagent containing 100 µM Amplex Red reagent and 0.2 U/mL HRP in 1× reaction buffer was added to each well. After incubation at room temperature in the dark for 30 min, the fluorescence intensity was detected by excitation at 540 nm and emission at 590 nm.

### 2.6. DCF Fluorescence Assay for the Detection of ROS

ROS levels were measured using 2′,7′-dichlorodihydrofluorescein diacetate (DCF-DA). PC cells (2 × 10^5^) were seeded in 24-well black-walled plates. After 80–85% confluence, cells were treated with His and/or Gem for 72 h. During the last 30 min of the treatment period, DCF-DA at 10 µM was added into the media and the cells were incubated at room temperature in the dark for 30 min. Cells were washed gently with HEPES buffer (2× times) and incubated in HEPES buffer for 15 min. The fluorescence was detected using a fluorescence plate reader by measuring the excitation and emission wavelengths at 492 nm and 517 nm, respectively.

### 2.7. Total Glutathione Assay

Total glutathione was determined colorimetrically using the glutathione assay kit (Cayman chemicals). Briefly, PC cells seeded in 100 mm Petri dishes were treated with His and/or Gem for 72 h. Cells were washed with PBS, sonicated, and cell lysates were prepared as per the manufacturer’s instructions. Samples were deproteinated by adding an equal volume of metaphosphoric acid and centrifuged at 2000× *g* for 2 min. Then, 50 µL of 4 M triethanolamine was added per mL of sample and vortexed immediately. The samples were diluted 1:20 and used for the measurement of total GSH. Briefly, 50 µL of the samples and standards were added in duplicate in each well of a 96-well plate, followed by 150 µL of assay cocktail, and the plate was incubated for 25 min in the dark. The intensity of color development was read at 410 nm.

### 2.8. Human and Animal Studies

De-identified human plasma samples were obtained from Nebraska Biobank. A human tissue array containing sections from pancreatic tumor and normal pancreas was obtained from US Biomax, Inc. De-identified normal and PC tissues were obtained from the UNMC tissue bank. All animal care procedures were carried out with approval from the Institutional Animal Care and Use Committee of the University of Nebraska Medical Center (Protocol Number: 18-034-04-FC). Six- to eight-week-old male and female, athymic nude mice (Strain: Hsd:Athymic Nude-Foxn1^nu^, Envigo, Indianapolis, IN, USA) and immunocompetent wild-type (mixed background) mice were used for the in vivo studies.

#### Mice and Diet

To assess the therapeutic efficacy of His in the presence or absence of Gem against PC, we used both the syngeneic and xenograft mouse models of PC by implanting murine PC cells (UN-KPC-960) and SW1990 cells, orthotopically, into the pancreases of wild-type and athymic mice, respectively. Six–eight-week-old male and female mice were used in the studies. The commercially available rodent diet (Research Diets Inc., New Brunswick, NJ, USA) provides about 0.46% to 0.6% His depending on the diet type. His as high as 1.875 g per kg body weight was used in mice in a previous study [23]. To study the effect of His in the presence or absence of Gem in altering the progression of a pancreatic tumor, we used a custom diet containing either normal His (0.6% His *wt*/*wt*, NH) or high His (1.8% His *wt*/*wt*, HH). The protein in the diet was provided by the AA mixture containing 19 different AAs except His. His was then added at 0.6% and 1.8% in NH and HH diets, respectively (Supplemental Table S2).

### 2.9. In Vivo Therapeutic Efficacy of His and/or Gem against PC

PC cells (0.25 × 10^6^/50 μL PBS) were injected into the head of the pancreas of mice. Eight to ten days post-surgery wounds were healed, and clips were removed. During this recovery period, the mice were on a normal chow diet. Ten days post-implantation, mice were switched to experimental diets containing NH or HH. Three days after starting the diet, mice were injected (I.P.) with Gem. We divided mice into four groups: (1) Control (NH), (2) His (HH), (3) Gem (NH + Gem), and (4) His + Gem (HH + Gem). Gem dosage as high as 25 mg/kg body weight twice weekly was used in previous studies [24,25]. To better assess the efficacy of the combination therapy, we administered Gem at 12.5 mg/kg body weight once weekly during the course of the experiment.

Two types of studies were conducted after the orthotopic transplantation of PC cells. In the first study, mouse survival was assessed in both syngeneic and xenograft models of PC. Mice were sacrificed when the tumor size was more than 2 cm or after veterinary alert. Survival data were analyzed with the Kaplan–Meier plot. In the second study, the effects of His and/or Gem on tumor growth and tumor characteristics were determined in the xenograft model. Body weight and tumor weight were recorded. Mice were sacrificed after 4 wk of treatment with His and/or Gem.

## 3. Analysis of Plasma His Levels

Human and mouse plasma samples were analyzed for AA profile using the HPLC system at the protein structure core facility at the University of Nebraska Medical Center (UNMC) and the hormone core laboratory at Vanderbilt University, respectively.

### 3.1. Histology

Hematoxylin and eosin (H + E) staining was performed on the human pancreatic cancer tissue array with our established protocol. For immunohistochemistry, mice were euthanized after 4 wk treatment with His and/or Gem. Pancreatic tissues were collected, fixed in 10% buffered formalin and paraffin-embedded. Pancreatic tissue was cut into 5 µm sections which were incubated with anti-Ki67 antibody (Abcam-Ab15580) overnight at 4 °C. After being washed, sections were incubated with a biotinylated secondary antibody (Vector IHC kit PK-4000) for 1 h at room temperature. Sections were then incubated with Vectastain ABC reagent (avidin-biotinylated horseradish peroxidase) for 30 min followed by incubation with peroxidase substrate (DAB; Vector SK-4100) until the desired color intensity developed. Sections were counterstained with hematoxylin. After counterstaining, sections were dehydrated and fixed with a toluene reagent. Images were captured using a Nikon Eclipse 80i inverted microscope.

### 3.2. Statistical Analysis

Results are presented as mean ± SEM. Overall mouse survival was calculated using the Kaplan–Meier curves and the difference was tested by the log-rank test. Statistical significance was determined by one-way analysis of variance followed by Tukey’s post hoc analysis. Graph-Pad Prism software was used to determine statistical significance (*p* < 0.05 was considered significant).

## 4. Results

### 4.1. Plasma His Levels Are Low in PC Patients

**Figure 1:**
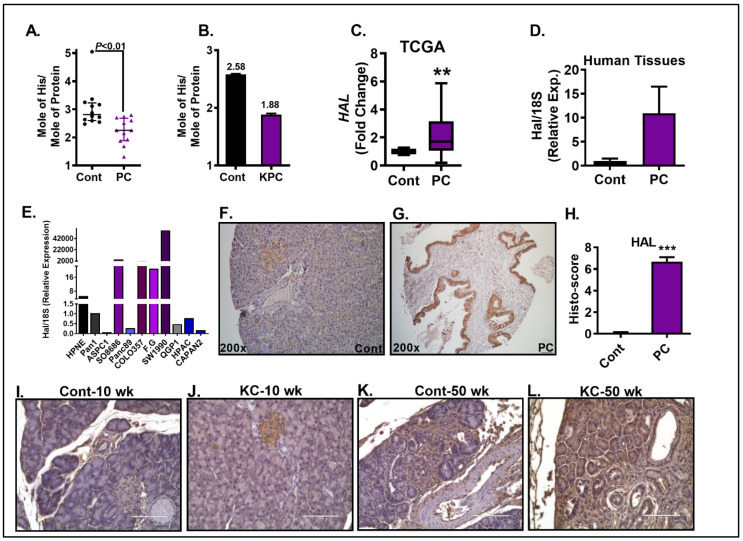
Analysis of circulating His and tissue HAL expression in pancreatic cancer. His levels in serum of PC patients and control subjects were measured (**A**). Analysis of His in serum of KPC mice and their controls (**B**). TCGA database shows a significant increase in *HAL* mRNA in PC patients compared to normal subjects (**C**). Hal mRNA level was higher in human PC tissue compared to normal tissue (**D**). Hal mRNA expression in different pancreatic cancer cell lines (**E**). HAL protein expression in normal (*n* = 47) (**F**) and pancreatic cancer tissues (*n* = 62) (**G**), and the corresponding histoscore (**H**). HAL immunostaining is higher in KC mice vs. age-matched controls at 10 (**I**,**J**) & 50 wk (**K**,**L**). Scale bar = 100 µm. ** *p* < 0.01, *** *p* < 0.001 vs. control.

Previous metabolomics analysis showed that the plasma-free AA profile was significantly altered in PC patients, with His levels being consistently lower compared to healthy subjects [12,13]. Our quantitative analysis of plasma-free AAs using an AA analyzer revealed that plasma His levels were in fact significantly lower (*p* < 0.01) in PC patients compared to controls (Figure 1A). The plasma levels of His along with other AAs in PC patients compared to normal subjects are shown in Supplemental Figure S1. To check if His levels were also altered in mice bearing pancreatic tumors, we analyzed the pooled plasma samples (3 samples per group) from KPC-tumor-bearing mice and their age-matched control. We noted a decrease in plasma His in KPC mice compared to control mice. Specifically, we detected 2.58 and 1.88 moles of His/mole of protein in control and KPC mice, respectively (Figure 1B).

### 4.2. The Expression of HAL Is Upregulated in PC

PC subjects exhibited low circulating His levels; therefore, we next wanted to check if the expression of HAL, the first rate-limiting enzyme in His catabolism, was altered in PC. Analysis of The Cancer Genome Atlas (TCGA) database showed a significant increase in HAL mRNA expression in PC compared to normal subjects (*p* < 0.01) (Figure 1C). We next analyzed HAL mRNA levels in the pancreas of normal versus PC patients. Our data revealed an upregulation of HAL mRNA in PC tissues compared to normal tissues (Figure 1D). *HAL* mRNA expression was also higher in many of the PC cell lines compared to normal HPNE cells (Figure 1E). Next, we performed immunohistochemical analysis on a human PC tissue array for HAL protein levels, which revealed a striking increase in HAL expression in PC compared to normal tissues (Figure 1F–H). In addition to the human PC tissues, the pancreatic sections from KC mice, a spontaneous model of PC exhibiting Kras mutation, also showed an increase in HAL staining in a time-dependent manner (Figure 1I–L). Taken together, these data provide novel evidence for the altered expression of HAL in PC.

### 4.3. His Exerts Cytotoxicity in PC Cells

**Figure 2:**
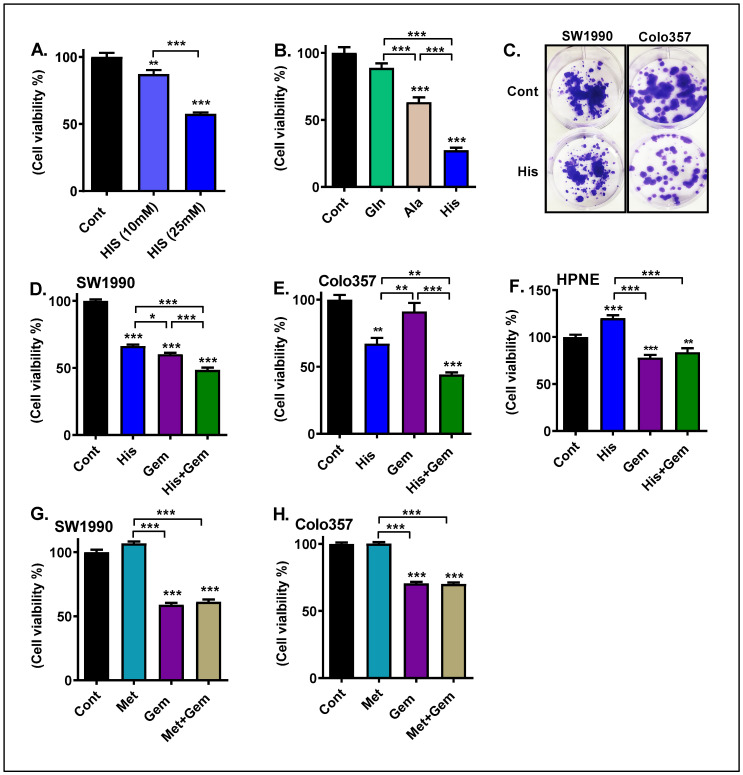
Percentage cell viability and colony forming assay in PC cells. SW1990 cells were cultured in amino acid (AA)-free DMEM F-12 media containing 5% FBS. Percentage cell viability was determined by MTT assay. Cells were treated with His at 10 mM and 25 mM for 72 h (**A**). Percentage cell viability of SW1990 cells treated with different AAs (25 mM) for 72 h (**B**). Colony formation assay was performed in His (5 mM)-treated SW1990 and Colo357 cells. A representative well from 3 wells per group is shown (**C**). Percentage cell viability of SW1990 cells treated with His (25 mM), Gem (10 µM), or His + Gem for 72 h (**D**). Percentage cell viability of Colo357 cells exposed to His and/or Gem (**E**). Percentage viability of HPNE cells treated with His (25 mM) and/or Gem (10 µM) for 72 h (**F**). Percentage viability of SW1990 (**G**) and Colo357 (**H**) cells was determined using the Presto blue reagent. Cells were treated with methionine (Met, 25 mM) and/or Gem (10 µM) for 72 h. Values are mean ± SEM of 7–12 samples per group. * *p* < 0.05, ** *p* < 0.01, *** *p* < 0.001 vs. control.

We next wanted to check whether His treatment alters the growth of PC cells in vitro. For the in vitro studies, we used the aggressive PC cells, SW1990 and Colo357, which exhibit an increased mRNA level of HAL. First, we analyzed the effects of His in altering the growth of SW1990 cells. We treated these cells at different concentrations of His and noted that His-induced cytotoxicity starting at a 10 mM concentration with a maximal effect at 25 mM concentration, as measured by MTT assay (Figure 2A). We next compared the effects of His with other AAs, including glutamine and alanine, on cell viability (Figure 2B). We noted that glutamine did not significantly alter cell viability; whereas, alanine treatment led to increased cell death. Importantly, His led to a profound decrease in cell viability. Our colony formation assay further confirmed that His inhibits the growth of PC cells (Figure 2C). Overall, our data indicate that His suppresses the growth of PC cells and is more effective in killing PC cells compared to other AAs like alanine.

### 4.4. His in Combination with Gem Treatment Exerted an Enhanced Cytotoxic Effect in SW1990 Cells

Gem response varies among different PC cell lines, and the IC50 of Gem ranges between 494 nM and 23.9 µM [26]. Our initial dose–response studies using Gem showed that Gem was effective starting at 1 µM but was more effective at a 10 µM concentration (not shown). Therefore, we performed further studies to determine the effect of His (25 mM) and/or Gem at 10 µM in modulating the viability of SW1990 cells cultured in AA-free media. As shown in Figure 2D, at 72 h of exposure, His and Gem alone led to a 34% and 40% reduction in cell viability, respectively. In combination, these two agents led to a 51% reduction compared to control (*p* < 0.001). Moreover, the cell viability was significantly lower in cells treated with His + Gem compared to Gem-only treated cells (*p* < 0.001). A similar trend with a lower magnitude difference was noted at 48 h of exposure (not shown). In separate experiments, PCs were cultured in regular DMEM containing AAs and 5% FBS and treated with His ± Gem. We noted a similar decline in cell viability in this condition (Supplemental Figure S2).

### 4.5. His in Combination with Gem Exerted an Enhanced Cytotoxic Effect in Colo357 Cells

As noted in Figure 2E, treatment with Gem alone did not significantly decrease cell viability in Colo357 cells even at 72 h of exposure, as measured by the MTT assay. This is in line with a previous report showing that Gem induced AKT phosphorylation, a pro-survival marker, and promoted Colo357 cell invasiveness [27]. In contrast, His-only led to a 33% decrease in cell viability (*p* < 0.001) compared to controls. When His was combined with Gem, there was a profound 56% decrease in cell viability (*p* < 0.001). These data suggest that combining Gem with His promotes cytotoxicity even in PC cells poorly responsive to Gem.

### 4.6. His Did Not Enhance the Cytotoxic Effect of Gem in Normal HPNE Cells

We next determined the effects of His ± Gem on normal pancreatic epithelial cells (HPNE cells), which are non-transformed immortalized cells. We noted that His rather exerted a growth-promoting effect in these cells even at 72 h of exposure (Figure 2F). Gem induced cytotoxicity in normal cells, but His + Gem did not increase it further. These data suggest the growth inhibitory effects of His are specific to PC cells.

### 4.7. Methionine Did Not Enhance the Cytotoxic Effect of GEM in PC Cells

We next wanted to check if the enhanced cytotoxic effect was exerted specifically by His or by other essential AAs. We treated Colo357 and SW1990 cells with methionine (Met), in the presence or absence of Gem. Interestingly, Met did not enhance the cytotoxic effect of Gem in SW1990 (Figure 2G) or Colo357 (Figure 2H) cells.

### 4.8. A Combination of His and Gem Leads to Increased ROS Production and Oxidative Stress

**Figure 3:**
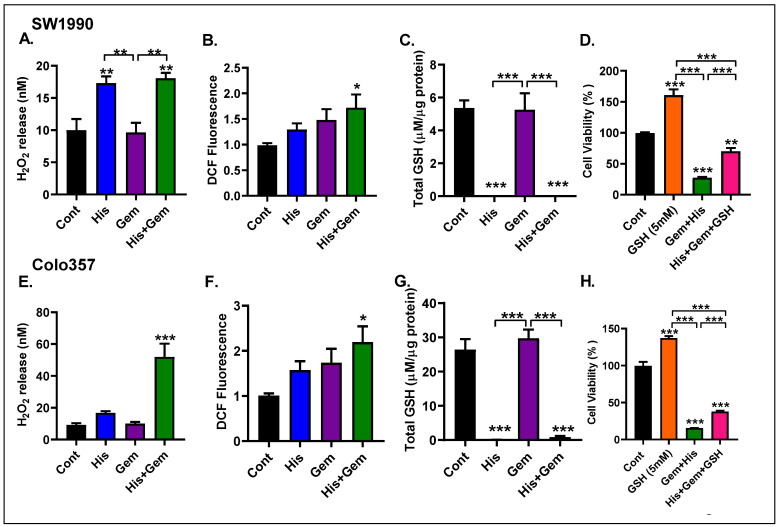
Effect of His and/or Gem on cellular oxidative stress. SW1990 and Colo357 cells were cultured in AA-free DMEM F-12 media containing 5% FBS and treated with His (25 mM) and/or Gem (10 µM). Hydrogen peroxide (H_2_O_2_) was measured in the media of SW1990 (**A**) and Colo357 cells (**E**) treated with His and/or Gem for 72 h. Cellular ROS levels were measured by DCF fluorescence in SW1990 (**B**) and Colo357 cells (**F**) treated with His and/or Gem for 24 h. Total glutathione (GSH) content was assessed in SW1990 (**C**) and Colo357 cells (**G**) after 72 h treatment with His and/or Gem. Cell viability was determined using the Presto Blue reagent in SW1990 (**D**) and Colo357 cells (**H**) treated for 72 h with Gem + His in the presence of absence of GSH (5 mM). * *p* < 0.05, ** *p* < 0.01, *** *p* < 0.001 vs. control.

Induction of ROS is an emerging approach for cancer therapy [Reviewed in [28]. To determine whether His + Gem-induced cytotoxicity is due to increased ROS generation and oxidative stress, we first determined the hydrogen peroxide (H_2_O_2_) content in the media. We noted a remarkable increase in H_2_O_2_ levels in media of SW1990 cells treated with His or His + Gem (*p* < 0.001) (Figure 3A). Of note, Gem by itself did not raise H_2_O_2_ levels. Analysis of DCF fluorescence, which detects many types of ROS, showed a trend towards an increase in Gem- and His-only treated cells and a significant increase (*p* < 0.05) in cells treated with a combination of both drugs compared to control (Figure 3B). Cellular levels of GSH, an antioxidant, was not altered in Gem-treated cells but were completely depleted in cells treated with His or His + Gem (*p* < 0.001) (Figure 3C). Interestingly, supplementing media with GSH partly protected against the cytotoxicity induced by a combination of His + Gem (Figure 3D). Together, these data suggest that adding His enhances the cytotoxic effects of Gem against PC cells by depleting GSH and increasing cellular oxidative stress. Regarding Colo357 cells, His-only led to a smaller increase in H_2_O_2_ release compared to SW1990 cells (Figure 3E). However, DCF fluorescence and GSH content showed a similar trend as seen in SW1990 cells upon His treatment (Figure 3F,G). As with SW1990 cells, a combination of His and Gem led to a significant increase in these markers of oxidative stress in Colo357 cells (Figure 3E–G), and GSH supplementation partially protected against His + Gem-induced cytotoxicity (Figure 3H).

### 4.9. His Treatment Leads to Its Intracellular Accumulation in PC Cells

**Figure 4:**
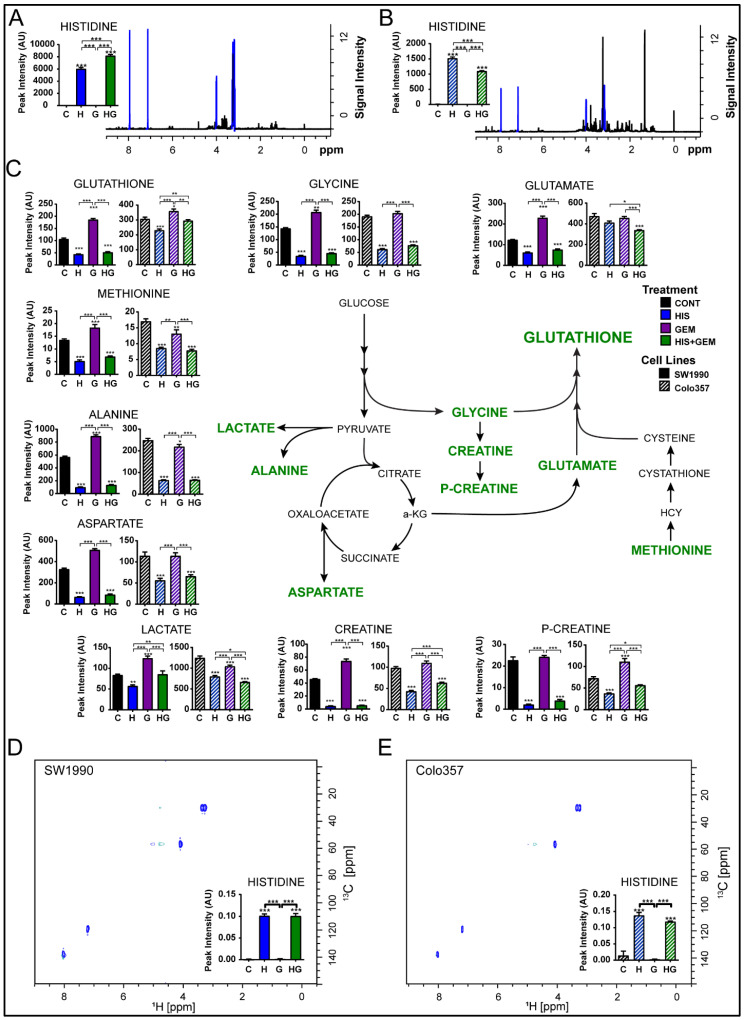
Metabolomics analysis in PC cells treated with His and/or Gem. Metabolite levels in SW1990 (solid bars) and Colo357 (hatched bars) cells in different treatment conditions have been plotted. SW1990 and Colo357 cells were treated with His (25 mM) and/or Gem (10 μM) in AA-free media containing 5% FBS (*n* = 6/group). A massive His accumulation was detected in SW1990 and Colo357 cells treated with His or His + Gem (**A**,**B**). His accumulation was associated with a depletion of glutathione and amino acids needed for glutathione synthesis, ammonia clearance, TCA cycle, and creatine synthesis (**C**). Accumulation of His in SW1990 and Colo357 cells was confirmed by treating cells with [^13^C, ^15^N]-His (**D**,**E**). * *p* < 0.05, ** *p* < 0.01, *** *p* < 0.001 vs. control.

We next performed metabolomics analysis to detect His-induced metabolic changes in SW1990 and Colo357 cells. His was not detectable in control and Gem-only treated cells. Alternatively, a profound increase in His accumulation was noted in His− and His + Gem-treated cells (Figure 4A,B). Metabolites directly associated with His metabolism were not detected in the cells. Multivariate analysis of both cell lines revealed His treatment significantly impacted cellular metabolism beyond His accumulation (Supplemental Figure S3). Interestingly, His treatment and accumulation were associated with a reduction in total glutathione and AAs supporting glutathione production (methionine, glycine, and glutamate). Moreover, AAs associated with the tricarboxylic acid (TCA) cycle (alanine, glutamate, and aspartate) as well as ammonia clearance (glutamate, alanine) were depleted. Furthermore, His treatment led to lactate depletion, a marker of anaerobic glycolysis (Figure 4C). In addition, creatine and phosphocreatine levels were significantly lower in His and His + Gem treated cells, an effect of glycine depletion and a contributing factor to ATP reduction. Taken together, these data suggested that His treatment leads to its accumulation in PC cells with a concomitant decrease in many AAs regulating oxidative stress and ATP production.

In a separate metabolomics experiment, we treated cells with stable isotope [^13^C,^15^N]-labeled His, and collected 2D ^1^H-^13^C HSQC spectra to characterize the utilization of His by PC cells. In line with the untargeted metabolomics analysis described above, our data showed a massive accumulation of His in both His− and His + Gem-treated cells (Figure 4D,E). However, we were unable to detect any metabolites derived from the [^13^C,^15^N]-labeled His in these experiments. These data indicate that His treatment leads to its increased uptake and accumulation, but no His-derived metabolites were detectable in PC cells.

### 4.10. A Combination of His and Gem Inhibited Pancreatic Tumor Growth In Vivo

**Figure 5:**
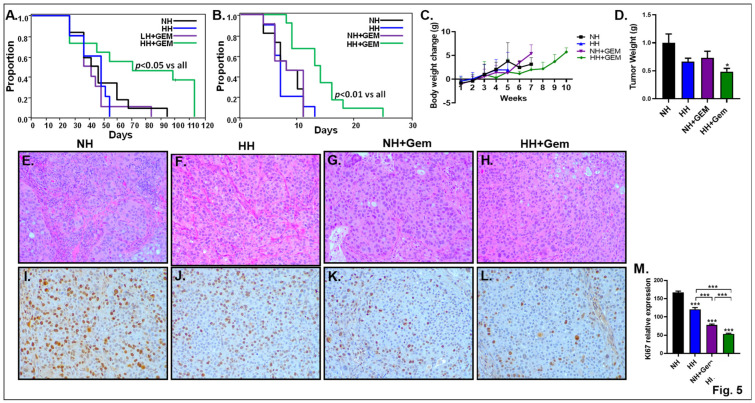
Athymic nude mice (xenograft model of PC) and wild-type mice (syngeneic model of PC) were implanted with SW-1990 cells and UN–KPC960 cells, respectively. Ten days post-implantation, mice were fed a diet containing normal His (NH) or high His (HH) with or without Gem treatment (12.5 mg/kg body weight). Mice were treated with Gem once a week for 4 wk. Survival curves for athymic nude mice (**A**) and wild-type mice (**B**) are shown. (*n* = 10–12/group). Body weight change was measured in tumor-bearing athymic nude mice during the course of the treatment (**C**). In a separate experiment, athymic nude mice implanted with SW-1990 cells were fed a diet containing normal His (NH) or high His (HH) with or without Gem treatment. After 4 weeks, mice were sacrificed at the same time point and tumor weight was assessed (*n* = 6–9/group), (**D**). H + E staining in pancreas sections is shown (**E**–**H**). Immunohistochemistry was performed for Ki67 in the pancreas (**I**–**L**). Images were captured at 20× magnification. Densitometry of Ki67 is shown (**M**). * *p* < 0.05 and *** *p* < 0.001 vs. NH.

The in vivo efficacy of His in combination with Gem was determined in mouse models of orthotopic pancreatic cancer. We evaluated the therapeutic potential of this combination on xenograft tumors produced by orthotopic implantation of human pancreatic cell lines (SW1990 cells) into athymic nude mice. In a separate experiment, we induced xenograft tumors by implanting syngeneic murine PC cell line (UN-KPC-960) into immunocompetent C57BL6/mixed background mice. We noted that the survival was not altered significantly in tumor-bearing athymic nude mice or wild-type mice that received treatment with His (HH-fed mice) or Gem only. Interestingly, a combination of His and Gem significantly increased survival in both athymic nude mice (*p* < 0.05) (Figure 5A) and wild-type mice (*p* < 0.01) (Figure 5B) exhibiting pancreatic tumors compared to controls. Athymic mice treated with His + Gem did not show a loss of body weight during the treatment period (Figure 5C). Analysis of the plasma AA profile showed a trend towards increased His plasma levels in HH diet-fed mice. Both Gem- and HH + Gem-treated mice showed a significant decrease in plasma His compared to the HH group (Supplemental Figure S4). Interestingly and in line with our metabolomics experiment in cultured PC cells, mice treated with a combination of His + Gem exhibited a significant decrease in plasma levels of glycine and methionine, compared to controls. Analysis of tumor weight showed a significant reduction in HH + Gem-treated athymic nude mice compared to the control (NH)-fed group (Figure 5D). These data clearly show that a combination of His and Gem is effective in attenuating tumor growth and improving the survival advantage of mice exhibiting xenograft tumors, without reducing body weight.

### 4.11. His in Combination with Gem Attenuates Ki67

We stained the pancreas sections with H + E and as shown in Figure 5E–H, mice in all groups showed pancreatic tumors, indicating that the tumor implants were successfully engrafted and were able to grow as orthotopic implants. Our in vitro data shows that inhibition of cell proliferation is one mechanism by which His inhibits the growth of PC cells (Figure 3C). Therefore, we stained the tumor sections for Ki67, a proliferation marker. We noted that the protein level of Ki67 was greatly attenuated in mice receiving a combination of His and Gem (Figure 5I–M).

## 5. Discussion

Herein, we provide novel evidence for the intimate relationship between His metabolism and PC. We demonstrate that circulating His levels were lower in both human subjects and mouse models of PC compared to controls. The expression of HAL, an enzyme involved in the His metabolic pathway, was greatly elevated in human and mouse pancreatic tumor tissue. His supplementation enhanced the efficacy of Gem in killing PC cells. Our metabolomics analysis clearly showed that His supplementation increased intracellular His accumulation, which was accompanied by a decrease in GSH levels and the depletion of AAs supporting GSH production. Accordingly, we further demonstrate that His-induced GSH depletion was associated with an increase in oxidative stress as evident from increased H_2_O_2_ production. Moreover, supplementation with GSH partially protected PC cells against His + Gem-induced cytotoxicity. In vivo studies further demonstrate that His in combination with Gem exerted an enhanced anticancer effect in orthotopic mouse models of PC in mice. Taken together, our data suggest that altered His metabolism is a feature of PC and that targeting His metabolism is an effective approach to improving the therapeutic benefit of anticancer drugs against this devastating disease.

We used SW1990 and Colo357 cells which have the propensity to develop resistance to Gem treatment. For example, SW1990 cells develop Gem resistance by increasing the proliferation of stem cell populations via ERK signaling [29]. Colo357 cells develop Gem resistance via upregulation of CXCR4 [27]. Of note, these two cell lines display a different genetic profile. For example, SW1990 cells exhibit a Kras mutation [30,31]; whereas, Colo357 cells have WT Kras [32,33,34]. However, evidence for a Kras mutation status in Colo357 cells is variable where some studies have indicated that Colo357 cells exhibited a Kras mutation [35,36]. Colo357 cells have also been reported to have a Braf mutation [34]. Using these two aggressive PC cell lines, we showed that His supplementation enhanced the therapeutic response to Gem. Our data also showed that the enhanced cytotoxicity was exerted by only a combination of His and Gem. Met, another essential AA, did not alter GEM-induced cytotoxicity in both SW1990 and Colo357 cells, indicating the specific effect of His in enhancing the response of PC cells to GEM treatment.

Regarding the mechanisms by which His leads to cell death, our data suggest that aberrant His uptake and accumulation over time leads to oxidative stress and cell death. We provide evidence that His treatment leads to a profound increase in H_2_O_2_ production in both SW1990 and Colo357 cells. Induction of ROS is one mechanism by which many anticancer drugs exert their therapeutic effects [37]. In particular, H_2_O_2_ plays an important role in oxidative-stress-induced cancer cell death [38,39]. In fact, an H_2_O_2_-generating system has emerged as an interesting anticancer alternative strategy to selectively kill cancer cells [38]. As cancer cells generate high concentrations of ROS and are under increased intrinsic oxidative stress, they might be more vulnerable to further oxidative insults produced by ROS-generating agents [40]. A previous study has shown that non-small-cell lung cancer cells resistant to anticancer treatment were sensitized in the presence of H_2_O_2_ [41]. Of note, His itself enhanced the cytotoxic effects of H_2_O_2_ in Chinese hamster ovary cells [42,43]. Moreover, a combination of His and H_2_O_2_ exerted an enhanced antimicrobial activity [44]. However, the direct effect of His on H_2_O_2_ production and subsequent cell death remains unknown and our study uncovered the novel role of His in generating H_2_O_2_ in PC cells. Moreover, our data provide clear evidence that His, but not Gem, increased H_2_O_2_ production in PC cells and suggest that His enhanced the anticancer effects of Gem by increasing H_2_O_2_. We also noted a concomitant reduction in cellular GSH pool upon His, but not Gem, treatment.

A question arises as to how His induces oxidative stress and promotes cytotoxicity in PC cells. Our metabolomics analysis clearly showed that His treatment led to its massive accumulation in PC cells. In normal cells, AA uptake is tightly regulated and depends on the need for individual AAs [45]. Our data suggest that PC cells exhibited an uncontrolled His uptake and accumulation. Interestingly, we did not detect any other His-derived metabolites; instead, we noticed that His accumulation was associated with a decrease in GSH levels with a concomitant depletion of certain AAs supporting GSH production. For example, our data showed that glycine and glutamate, which are direct components of GSH, were depleted in cells treated with His and His + Gem. Moreover, metabolites upstream of GSH were significantly affected by His treatment. For example, the methionine cycle generates homocysteine, a precursor for cysteine, which is another AA in GSH. We noted that methionine was also depleted in cells upon His supplementation. Thus, depletion of GSH due to the depletion of AAs needed for GSH production is a likely mechanism for His-induced oxidative stress and cell death.

It should be noted that these AAs which are involved in GSH synthesis also have other functions including the regulation of cell proliferation and survival. For example, glycine has an important role in promoting the growth of PC cells via two mechanisms: (1) by increasing the antioxidant pool, and (2) through its participation in one-carbon metabolism that contributes to nucleotide biosynthesis [46,47]. Similarly, glutamate is an important anaplerotic substrate for the TCA cycle and is a detoxifying agent for free ammonia. The methionine cycle plays an important role in maintaining the levels of tetrahydrofolate (THF_4_), which is central to one-carbon metabolism that promotes nucleotide synthesis and cancer cell proliferation. Therefore, the role of His in altering these other cell survival mechanisms cannot be ruled out.

Our metabolomics analysis also showed that the levels of creatine and phosphocreatine were reduced in cells treated with His or His + Gem. Glycine and methionine which are reduced upon His treatment, are important contributors to creatine synthesis. Emerging evidence suggests that creatine promotes cancer metastasis [48] and therefore, a decrease in creatine upon His treatment can also account for the improved therapeutic benefit of the combination of His + Gem. Taken together, our metabolomics data suggest that His accumulation leads to the depletion of many AAs, which are needed to reduce oxidative stress and support cell survival. This notion is supported by a previous report showing that the AA pool was significantly decreased in MV4-11 leukemic cells treated with a combination of CUDC-907 and gilteritinib, with a concomitant reduction in cell viability [49].

Dietary intervention is highly popular among cancer patients. Mounting evidence suggests that AA restrictions are potential strategies for cancer treatment. For example, studies have shown that dietary restriction of some AAs, including methionine [50], serine [51], and serine + glycine [52,53] inhibits tumor growth. While deprivation of some AAs inhibits tumor growth, our in vivo data show that dietary supplementation with His enhances the anticancer effects of Gem against PC. We noted a reduction in tumor size and an improvement in the survival of mice treated with this combination. Our data align with a recent study that showed that His enhanced the sensitivity of cancer cells to methotrexate [16]. His is one of the least abundant AA in plant-based foods [54]. Although meat and meat products have a higher histidine content [55], they are also rich in fat content and therefore their consumption should be limited. The requirement of specific AAs increases in certain physiological and pathological conditions. Thus, it is reasonable to speculate that His requirement may be increased to inhibit the progression of PC and that His supplementation is a promising adjuvant therapy that enhances the anticancer effects of Gem. Although HAL was over-expressed in PC cells and tissues, it is still unclear whether HAL is an oncogene or a tumor suppressor. Further studies using HAL siRNA will provide a better understanding on its role in mediating His effects on cell viability, ROS production, and other cellular responses.

## 6. Conclusions

Taken together, our data show that His uptake and/or metabolism is aberrantly regulated in PC. His treatment leads to a massive His accumulation in cells, which leads to an increase in H_2_O_2_ and the depletion of cellular GSH. Furthermore, His accumulation is accompanied by a reduction in AAs which contribute to GSH synthesis and/or cell survival. The increased accumulation of His enhances the therapy response to Gem by increasing H_2_O_2_ and depleting GSH which, in turn, results in cytotoxicity. Our study provides a mechanistic rationale for the consideration of His supplementation as a safe nutritional approach to improving the anticancer effects of chemotherapeutic drugs.

## Data Availability

The data presented in this study are available in this article (and supplementary material).

## Supplementary Materials

The following supporting information can be downloaded at: https://www.mdpi.com/article/10.3390/cancers15092593/s1. Table S1. Reagents and research tools. Table S2. Diet Composition. Figure S1. Amino acid profile in human plasma samples. Control group (*n* = 12) & Pancreatic Cancer (PC) group (*n* = 12). Histidine and threonine levels were significantly reduced in PC group while glutamine level was increased significantly. Other amino acids levels were not significantly altered. * *p* < 0.05, and ** *p* < 0.01. Figure S2. Cell viability assay was performed in SW1990 (A) and Colo357 (B) using the Presto Blue cell viability reagent. PC cells were treated with His in the presence or absence of Gem using regular DMEM with 5% FBS. His + Gem treated group showed the highest cytotoxicity compared to other groups in both cell lines. *** *p* < 0.001. Figure S3. Multivariate models for 1H 1D NMR Metabolomics Analysis of (A) SW1990 and (B) Colo357 cell lines. Left panels show principal component analysis (PCA) of the four treatment groups, control (red), Gem (green), His (yellow), and His + Gem (blue). Dendrogram models display the Mahalanobis distance as *p* values. The right panels show orthogonal projection to latent structures (OPLS) back-scaled loading plots of Cont v His and Cont v His + Gem for the respective cell lines. Annotated metabolites as follows (1) lactate, (2) alanine, (3) glutamine, (4) creatine, (5) histidine, (6) choline, (7) phosphocholine, and (8) glycine. Figure S4. Amino acid profile in mouse plasma samples. Orthotopic transplantation of the PC cell line SW1990 was done in the pancreas of athymic nude mice. Mice were divided into four groups: Normal Histidine (NH), High histidine (HH), Normal His + Gem (NH + Gem) and High histidine + Gem (HH + Gem). *n* = 6–8/group. Some of the amino acids levels were significantly altered. * *p* < 0.05 and ** *p* < 0.01.
