# Supplementary material for: Histidine Enhances the Anticancer Effect of Gemcitabine against Pancreatic Cancer via Disruption of Amino Acid Homeostasis and Oxidant—Antioxidant Balance
Source: Cancers (Basel). 2023 May 3;15(9):2593. doi: 10.3390/cancers15092593 (PMC10177467; doi:10.3390/cancers15092593)
Supplement: Supplementary file 1 [file cancers-15-02593-s001.zip › cancers-2213680-supplementary.pdf]

List of Abbreviations

1D One dimensional

2D Two dimensional

AA Amino acids

a-KG α-ketoglutarate

Ala Alanine

Arg Arginine

Asn Asparagine

ATP Adenosine 5′-triphosphate

BMRB Biological magnetic resonance bank

Citr Citrulline

CONT Control

$CXCR_4$ C-X-C chemokine receptor type 4

Cys Cysteine

D_2O Deuterium Oxide

DCF-DA 2',7' Dichlorodihydrofluorescein diacetate

DMEM Dulbecco's modified eagle medium

DMG Dimethyl glycine

ERK Extracellular signal-regulated kinase

FBS Fetal bovine serum

GEM Gemcitabine

Gln Glutamine

Glu Glutamate

Gly Glycine

GSH Glutathione

H&E Hematoxylin and eosin

H_2O_2 Hydrogen peroxide

HAL Histidine ammonia lyase

HCY Homocysteine

HH High histidine

HIS Histidine

HMDB Human metabolome database

HPLC High performance liquid chromatography

HPNE Normal pancreatic epithelial cells

HSQC Heteronuclear single-quantum correlation spectroscopy

Ile Isoleucine

K-ras Kirsten rat sarcoma viral oncogene homolog

Leu Leucine

Lys Lysine

MEM Minimum essential medium

Met Methionine

MTT (3-(4,5-dimethylthiazol-2-yl)-2,5-diphenyltetrazolium bromide)

NH Normal histidine

NMR Nuclear magnetic resonance

Orn Ornithine

PC Pancreatic cancer

PCA Principle component analysis

Phe Phenylalanine

PI Propidium iodide

Pro Proline

ROS Reactive oxygen species

Ser Serine

TCA cycle Tricarboxylic acid cycle

TCGA The cancer genome atlas

THF_4 Tetrahydrofolate

Thr Threonine

TMSP 3-(Trimethylsilyl)propionic-2,2,3,3-d4 acid sodium salt

Trp Tryptophan

Tyr Tyrosine

Val Valine

Supplemental Table S1. Reagents and research tools

REAGENT/ RESOURCE	SOURCE	CATALOG#
**Chemicals/Reagents/Kits**		
Dulbecco's Modified Eagle's Medium	Hyclone	SH30243.01
DMEM F-12 Amino Acid Free Medium	US Bilologicals	9807-09
L-Histidine	Sigma-Aldrich	H6034
L-Methionine	Sigma-Aldrich	M9625
$^{13}C^{15}N$ Histidine	Sigma-Aldrich	608009
Thiazolyl blue tetrazolium bromide (MTT assay)	Sigma-Aldrich	M2128
Presto blue	Thermo fisher scientific	A13261
Annexin-V/Cy™5 and propidium iodide	BD Biosciences	BD 559934
Amplex red hydrogen peroxide assay kit	Invitrogen	A22188
2',7'- dichlorodihydrofluorescin diacetate (DCF)	Invitrogen	D399
Glutathione assay kit	Cayman Chemical	703002
**Animals/Cell-lines/others**		
Hsd:Athymic Nude-Foxn1^{nu}	Envigo^{++++}	069
SW1990	ATCC	
Colo357	ATCC	
HPNE	Dr. Ouelette (UNMC)	
UN-KPC-960	Established at UNMC	
De-identified human plasma	Nebraska Biobank	
De-identified human pancreatic tissues	UNMC tissue bank	
Human pancreatic tissue array	Biomax	PA2081bt
Normal histidine diet	Research Diets Inc.	A10021B
High histidine diet	Research Diets Inc.	A19062801

Supplemental Table S2. Diet Composition

	(Normal Histidine Diet)		(High Histidine Diet)	
**Ingredients (gm)**	**gm%**	**kcal%**	**gm%**	**kcal%**
Protein	17	18	18	19
Carbohydrate	69	70	67	70
Fat	5	12	5	12
Total		100		100

	0.6 % Histidine diet (Normal Histidine Diet)		1.8 % Histidine diet (High Histidine Diet)	
**Ingredients (gm)**	**gm**	**kcal%**	**gm**	**kcal%**
L-Arginine	10	40	10	40
L-Histidine-HCl-H2O	6	24	18	72
L-Isoleucine	8	32	8	32
L-Leucine	12	48	12	48
L-Lysine-HCl	14	56	14	56
L-Methionine	6	24	6	24
L-Phenylalanine	8	32	8	32
L-Threonine	8	32	8	32
L-Tryptophan	2	8	2	8
L-Valine	8	32	8	32
L-Alanine	10	40	10	40
L-Asparagine-H2O	5	20	5	20
L-Aspartate	10	40	10	40
L-Cystine	4	16	4	16
L-Glutamic Acid	30	120	30	120
L-Glutamine	5	20	5	20
Glycine	10	40	10	40
L-Proline	5	20	5	20
L-Serine	5	20	5	20
L-Tyrosine	4	16	4	16
Corn Starch	550.5	2202	538.5	2154
Maltodextrin 10	125	500	125	500
Cellulose	50	0	50	0
Corn Oil	50	450	50	450
Mineral Mix S10001	35	0	35	0
Sodium Bicarbonate	7.5	0	7.5	0
Vitamin Mix V10001	10	40	10	40
Choline Bitrartrate	2	0	2	0
Blue Dye, FD&C #1	0.05	0	0	0
Red Dye, FD&C #40	0	0	0.05	0

**Total**	**1000.05**	**3872**	**1000.05**	**3872**

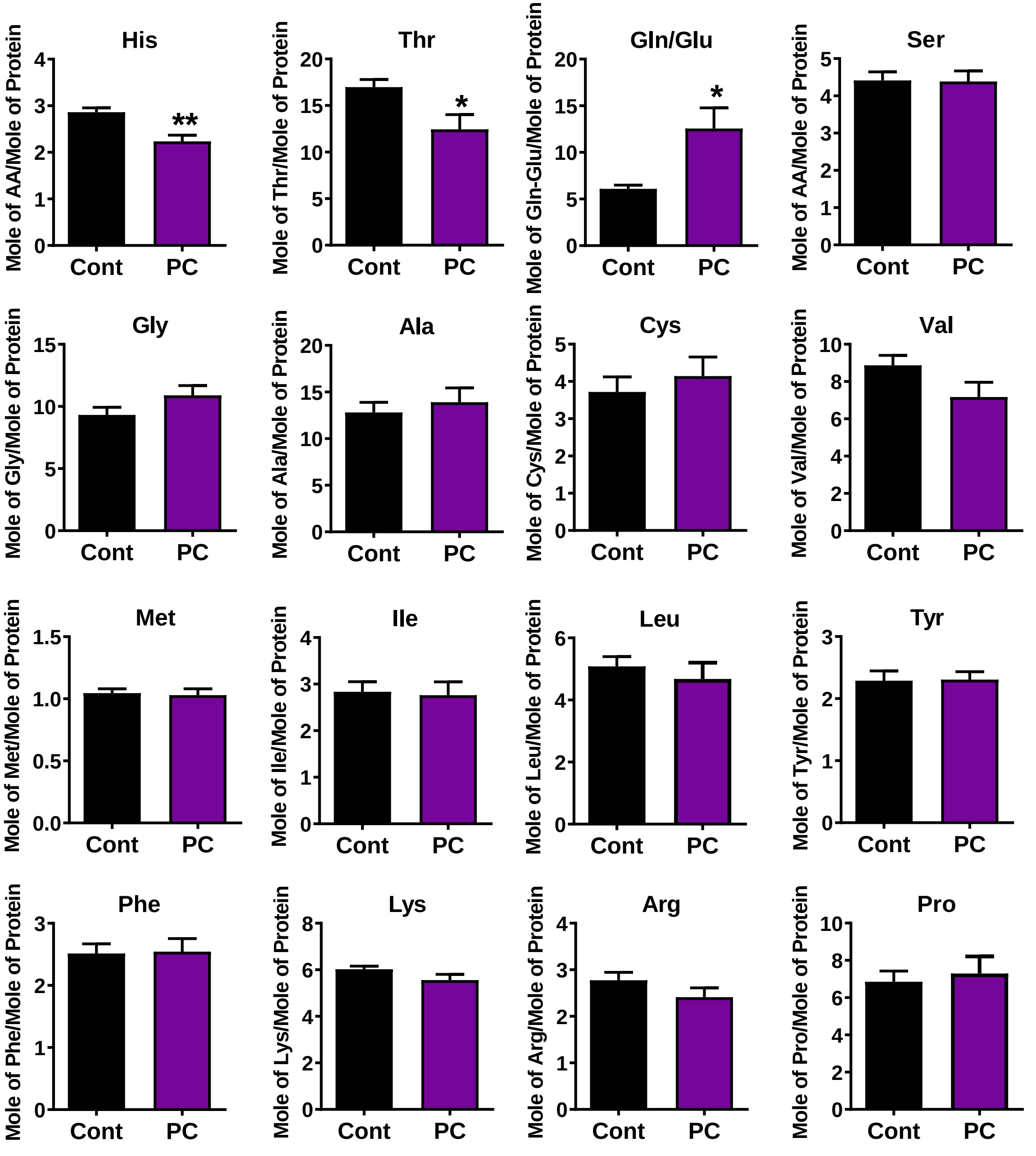

Supplemental Figure S1. Amino acid profile in human plasma samples. Control group (n=12) & Pancreatic Cancer (PC) group (n=12). Histidine and threonine levels were significantly reduced in PC group while glutamine level was increased significantly. Other amino acids levels were not significantly altered. $*P<0.05$, and $**P<0.01$

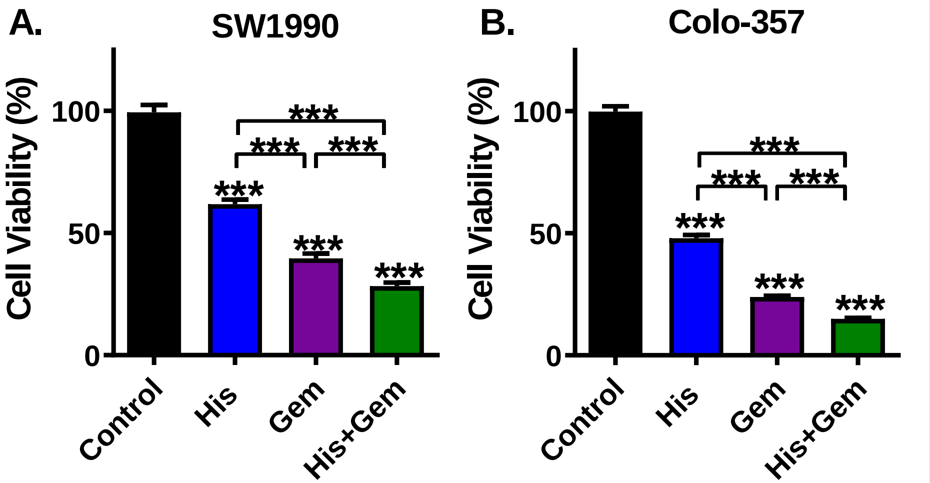

Supplemental Figure S2. Cell viability assay was performed in SW1990 (A) and Colo357 (B) using the Presto Blue cell viability reagent. PC cells were treated with His in the presence or absence of Gem using regular DMEM with 5% FBS. His+Gem treated group showed the highest cytotoxicity compared to other groups in both cell lines. ****P*<0.001

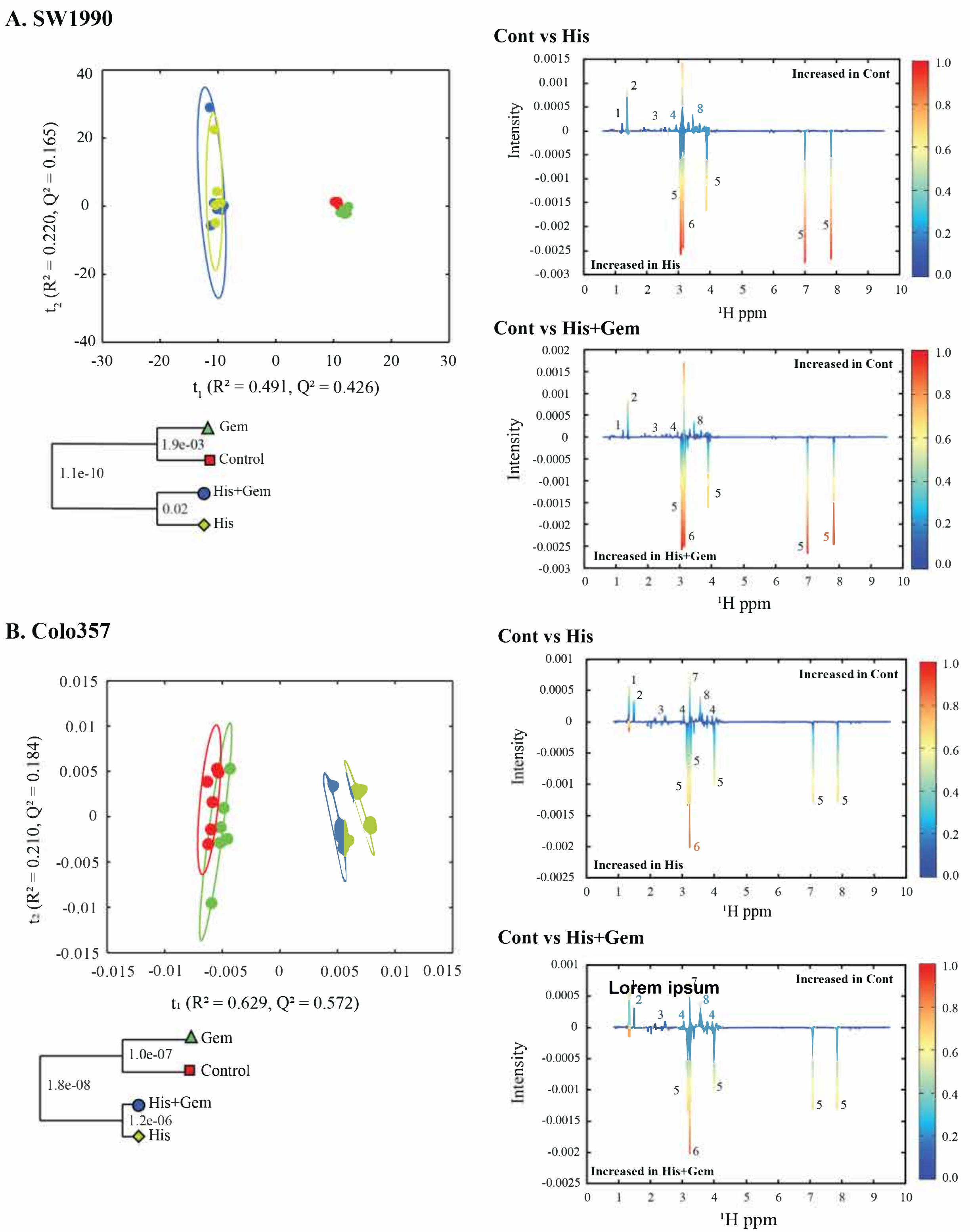

Supplemental Figure S3. Multivariate Models for 1H 1D NMR Metabolomics Analysis of (A) SW1990 and (B) Colo357 cell lines. Left panels show principal component analysis (PCA) of the four treatment groups control (red), Gem (green), His (yellow), and His+Gem (blue). Dendrogram models display the Mahalanobis distance as p-values. The right panels show orthogonal projection to latent structures (OPLS) back-scaled loading plots of Cont v His and Cont v His+Gem for the respective cell lines. Annotated Metabolites as follows (1) lactate, (2) alanine, (3) glutamine, (4) creatine, (5) histidine, (6) choline, (7) phosphocholine, (8) glycine.

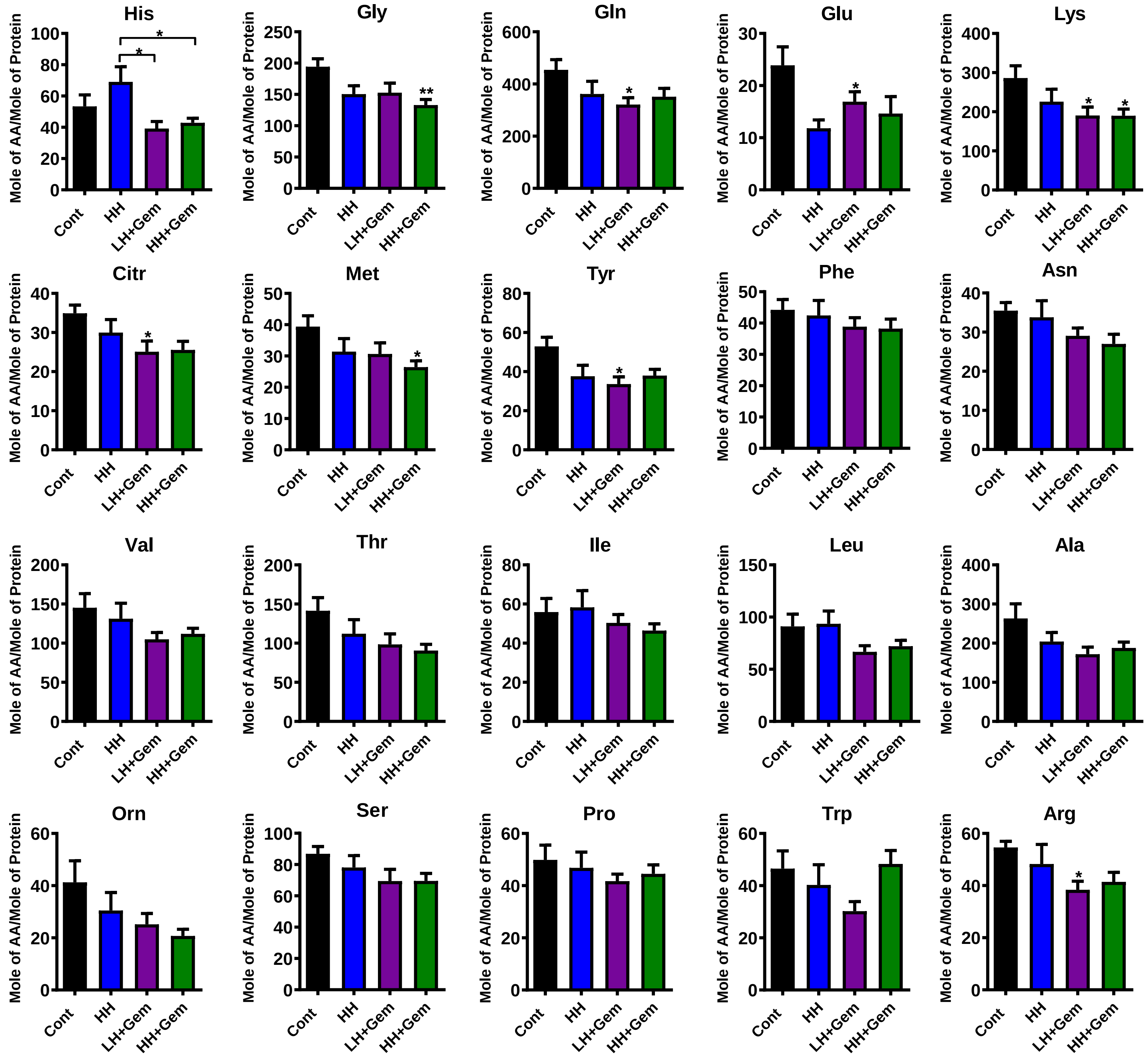

Supplemental Figure S4. Amino acid profile in mouse plasma samples. Orthotopic transplantation of the PC cell line SW1990 was done in the pancreas of athymic nude mice. Mice were divided into four groups: Normal Histidine (NH), High histidine (HH), Normal His + Gem (NH+Gem) and High histidine + Gem (HH+Gem). N=6-8/group. Some of the amino acids levels were significantly altered. *P<0.05 and **P<0.01
